# Suction Ureteral Access Sheaths During Flexible Ureteroscopy for Renal Stones: A Prospective Study and Cost Analysis

**DOI:** 10.7759/cureus.98616

**Published:** 2025-12-07

**Authors:** Fraser Barbour, Sidney D Parker, Jenny Koo Ng, Alastair D'Oyley, David Bryant, Ameet Patel, Ben Jenkins, Stuart Mccracken, Ken MacKenzie

**Affiliations:** 1 Urology, Sunderland Royal Hospital, Sunderland, GBR

**Keywords:** endourology, flexible ureteroscopy, nd:yag laser, renal stone disease, ureteral access sheath, ureterorenoscopy, urology, urology stone basket, urology surgery, urology technology

## Abstract

Background

During flexible ureteroscopy (fURS), ureteral access sheaths (UASs) are used to protect the fURS scope and reduce intrarenal pressure. UASs with suction functionality are increasingly used during fURS, with purported benefits including faster stone clearance, the ability to treat larger stones, reduced intrarenal pressure (potentially decreasing infections), and cost savings due to less frequent use of expensive baskets. This single-center study aimed to compare outcomes of fURS cases using suction UASs (S-UASs) versus traditional UASs (T-UASs) and to assess potential cost savings.

Methods

Data were collected prospectively from the first 50 fURS cases performed for stone disease in adult patients using the ClearPetra S-UAS (August 2024 to February 2025) and compared with 50 fURS cases using T-UAS prior to the availability of S-UAS (April to July 2024). From August 2024, the choice of S-UAS, T-UAS, or no access sheath during fURS was at the discretion of the operating surgeon. Qualitative data were compared using Fisher’s exact test or chi-squared test as appropriate. Quantitative data were compared using the Mann-Whitney U test. p-Values <0.05 were considered statistically significant. Costs used in the analysis were based on this single center’s equipment prices.

Results

Baskets were used significantly less in the S-UAS group (p < 0.001), resulting in estimated cost savings of £115 per fURS, totaling £5,750 over 50 cases. There were no statistically significant differences between the S-UAS and T-UAS groups in rates of persistent stones on follow-up imaging at three and six months, reintervention, or complications (p > 0.05). Median operative time was longer in the S-UAS group, but this did not reach statistical significance (p = 0.099). The S-UAS group had higher rates of multiple stones, partial staghorn stones, stones larger than 2 cm, and bilateral fURS, as well as a lower rate of pre-stenting; however, none of these differences reached statistical significance (p > 0.05).

Conclusions

S-UAS usage during fURS for renal stones resulted in significantly less basket usage and associated cost savings. Rates of persistent fragments, reintervention, and complications were similar between the S-UAS and T-UAS groups. Operative time appeared longer in the S-UAS group, although this was not statistically significant. A large randomized controlled trial with a predefined follow-up imaging schedule, specifically evaluating whether S-UAS reduces reintervention rates compared with T-UAS following fURS, would be valuable to further investigate its effectiveness.

## Introduction

Kidney stones are a common condition, estimated to affect approximately 10% of the UK population at some point in their lifetimes [1,2]. Management options include conservative measures, extracorporeal shockwave lithotripsy (ESWL), flexible ureteroscopy (fURS), percutaneous nephrolithotomy (PCNL), or, rarely, open surgery [3].

The European Association of Urology (EAU) 2025 guidelines [4] recommend fURS as the first-line treatment for renal stones of 2 cm or less. Using fURS for these cases results in fewer side effects compared with PCNL and improved stone clearance rates compared with ESWL [4-6]. PCNL is recommended for stones larger than 2 cm [4,7], while ESWL or fURS are advised for renal stones smaller than 1 cm and for proximal ureteric stones [4,8].

Ureteral access sheaths (UASs) are used in most fURS cases to facilitate repeated access to the kidney with the flexible ureteroscope, improve drainage and irrigation flow (enhancing visibility), and reduce intrarenal pressures [9,10]. Suction UASs (S-UASs) have been developed with purported benefits, including faster stone clearance, reduced intrarenal pressure (potentially lowering infection risk), and cost savings due to less frequent use of expensive baskets [11-13]. S-UASs may also allow fURS to be used for larger stones that would traditionally be treated with PCNL, as suction improves visibility despite the debris generated from lasering large stones [14-16]. Baskets may still be required in some cases, such as when repositioning stones in difficult-to-access locations like lower pole calyces with tight infundibulopelvic angles [11].

Elevated intrarenal pressures during fURS have been associated with increased risk of renal damage and postoperative UTIs [17,18]. EAU guidelines recommend measures to reduce intrarenal pressure during fURS, as high pressures predispose patients to complications [4]. S-UASs aim to lower intrarenal pressure by enhancing drainage during the procedure, which may reduce the incidence of postoperative complications, including UTIs [11,13].

Some studies of S-UASs have shown promising results, suggesting they may lead to higher stone-free rates and lower infection rates [12,13]. However, findings have been mixed regarding whether S-UASs reduce reintervention rates or shorten operative time [12,19,20]. This study aims to evaluate whether S-UASs improve renal stone clearance during fURS and whether they lead to cost savings through reduced use of expensive baskets.

## Materials and methods

This study was conducted in the Urology Department at a large acute hospital. It was a single-center comparative cohort study with prospective and retrospective arms. The sample size was calculated with a power of 80% and a two-sided confidence level of 95%, using the stone-free rates of 78% for S-UAS and 48% for traditional UAS (T-UAS) from Liu et al.’s meta-analysis [12], which suggested a minimum of 39 patients per group.

The prospective group comprised the first 50 fURS cases performed using the S-UAS from August 2024 to February 2025. The S-UAS used in this study was the ClearPetra access sheath. From August 2024, the choice of using an S-UAS, a T-UAS, or no access sheath was left to the discretion of the operating surgeon, as was the choice of access sheath diameter. The retrospective group included 50 fURS cases using T-UAS performed prior to the availability of S-UAS, from April to July 2024. Hospital operating theater records were used to identify cases for inclusion. Once identified, data were collected from hospital electronic records, including patient demographics, prior stenting on the side of fURS, stone characteristics on preoperative CT scans, intraoperative findings from operation notes, follow-up data including imaging and reinterventions, and stone analysis. Demographics collected included age, sex, American Society of Anesthesiologists (ASA) Physical Classification System grade, and previous history of renal stone disease.

Adults aged 18 years or older undergoing fURS for renal tract stones at this single center who had an access sheath used during surgery were included in the study. Exclusion criteria were cases in which the preoperative CT scan was unavailable for analysis, fURS performed for reasons other than renal tract stones, or fURS during which no stones or only matrix stones were found.

The primary endpoint of the study was to evaluate whether the rate of persistent stone fragments on follow-up imaging was lower with S-UAS compared to T-UAS at three and six months post-fURS. Secondary endpoints included assessing whether S-UAS was associated with shorter operative times, fewer complications, lower reintervention rates for persistent fragments, and reduced procedure costs due to less basket usage. Cost analysis was based on the hospital’s equipment prices; other factors, including staff and theater costs, were assumed to be identical. Reintervention was defined as any stone procedure performed for persistent fragments, including fURS, ESWL, or PCNL.

Continuous variables are presented as median with IQR, and categorical variables as n (%). Data were evaluated according to variable type using standard statistical methods. Qualitative data were compared using Fisher’s exact test or the chi-squared test, as appropriate. Quantitative data were compared using the Mann-Whitney U test. p-Values <0.05 were considered statistically significant.

## Results

Data were collected on a total of 100 patients: the first 50 fURS cases for stones using the ClearPetra S-UAS at this single center (August 2024 to February 2025) and 50 fURS cases for stones using T-UAS (prior to the availability of S-UAS) from April 2024 to July 2024. Patient demographics, stone type, and preoperative stone burden are summarized in Table 1.

**Table 1:** Patient demographics and stone characteristics ^*^ Mann-Whitney U test ^**^ Chi-squared test ^†^ Fisher’s exact test p-Values <0.05 were considered statistically significant. ASA, American Society of Anesthesiologists; S-UAS, suction ureteral access sheath; T-UAS, traditional ureteral access sheath

Characteristic	S-UAS group (n = 50)	T-UAS group (n = 50)	p-Value
Median age (year)	67 (IQR: 56-74)	66 (IQR: 51-73)	0.37^*^
Sex	29 males (58%), 21 females (42%)	27 males (54%), 23 females (46%)	0.84^†^
History of previous stones	32 (64%)	30 (60%)	0.84^†^
Pre-stented	33 (66%)	38 (76%)	0.38^†^
ASA grade			0.12^†^
ASA I	0	3 (6%)	
ASA II	22 (44%)	24 (48%)	
ASA III	25 (50%)	23 (46%)	
ASA IV	3 (6%)	0	
Median largest stone size (mm)	10 (IQR: 8-12)	10 (IQR: 7-12)	0.97^*^
Largest stone size			0.38^†^
<10 mm	24 (48%)	22 (44%)	
10-20 mm	24 (48%)	28 (56%)	
>20 mm	2 (4%)	0	
Median density of the largest stone (HU)	807 (IQR: 602-1106)	838 (IQR: 568-979)	0.65^*^
Partial staghorn stones	3 (6%)	1 (2%)	0.62^†^
Stone location			0.56^**^
Multiple locations	26 (52%)	21 (42%)	
Upper pole only	3 (6%)	1 (2%)	
Renal pelvis/interpolar only	6 (12%)	8 (16%)	
Lower pole only	12 (24%)	14 (28%)	
Ureter only	3 (6%)	6 (12%)	

Median ages were similar between the two groups: 67 years (IQR: 56-74) in the S-UAS group and 66 years (IQR: 51-73) in the T-UAS group (p = 0.37). Gender distribution was also similar: 29 males (58%) and 21 females (42%) in the S-UAS group versus 27 males (54%) and 23 females (46%) in the T-UAS group (p = 0.84). The proportion of patients with a history of renal stones was comparable: 32 (64%) in the S-UAS group and 30 (60%) in the T-UAS group (p = 0.84). The rate of pre-stenting was slightly lower in the S-UAS group at 33 (66%) compared with 38 (76%) in the T-UAS group, but this difference was not statistically significant (p = 0.38).

There was no statistically significant difference in ASA grade between the groups (p = 0.12); however, three patients (6%) in the S-UAS group had an ASA grade of IV, compared with none in the T-UAS group. The median largest stone size was 10 mm in both groups, and the distribution of stones <10 mm, 10-20 mm, and >20 mm was not statistically significant (p = 0.38). Notably, two patients in the S-UAS group had stones >20 mm, compared with none in the T-UAS group. Median density of the largest stone, measured in HU, was similar: 807 HU (IQR: 602-1106) in the S-UAS group and 838 HU (IQR: 568-979) in the T-UAS group (p = 0.65). Three patients (6%) in the S-UAS group had partial staghorn stones, compared with one patient (2%) in the T-UAS group, though this difference was not statistically significant (p = 0.62).

Stone location distribution was similar between groups (p = 0.56). In both groups, the most common location was multiple sites: 26 patients (52%) in the S-UAS group and 21 patients (42%) in the T-UAS group.

Table 2 presents the intraoperative findings from the fURS cases. In the S-UAS group, 37 patients (74%) had multiple stones, and 11 patients (22%) had more than five stones, compared with 31 patients (62%) and six patients (12%), respectively, in the T-UAS group; however, these differences were not statistically significant (p = 0.28 and p = 0.29). Bilateral fURS was performed in four patients (8%) in the S-UAS group, compared with two patients (4%) in the T-UAS group, which was also not statistically significant (p = 0.68).

**Table 2:** Intraoperative findings ^* ^Mann-Whitney U test ^**^ Chi-squared test ^†^ Fisher’s exact test p-Values <0.05 were considered statistically significant. fURS, flexible ureteroscopy; S-UAS, suction ureteral access sheath; T-UAS, traditional ureteral access sheath

Characteristic	S-UAS group (n = 50)	T-UAS group (n = 50)	p-Value
Multiple stones found intraoperatively	37 (74%)	31 (62%)	0.28^†^
>5 stones	11 (22%)	6 (12%)	0.29^†^
Bilateral fURS	4 (8%)	2 (4%)	0.68^†^
Median operative time (minutes)	75 (IQR: 63-97)	67 (IQR: 56-91)	0.099^*^
Basket used	14 (28%)	45 (90%)	<0.001^†^
Post-op stenting			0.84^†^
Stent (strings off)	33 (66%)	31 (62%)	
Stent (strings on)	17 (34%)	18 (36%)	
No stent	0	1 (2%)	

Median operative time was longer in the S-UAS group at 75 minutes (IQR: 63-97) compared with 67 minutes (IQR: 56-91) in the T-UAS group, although this difference did not reach statistical significance (p = 0.099). Baskets were used significantly less frequently in the S-UAS group (14 patients, 28%) than in the T-UAS group (45 patients, 90%) (p < 0.001). There was no significant difference between groups in the method of postoperative ureteric stenting (p = 0.84).

Table 3 shows the follow-up results after fURS. Postoperative stone analysis revealed no significant difference in stone types between the two groups (p = 0.58). Calcium oxalate stones were the most common, found in 21 patients (42%) in the S-UAS group and 26 patients (52%) in the T-UAS group. Stone analysis was not completed in five (10%) S-UAS cases and four (8%) T-UAS cases.

**Table 3:** Follow-up results after fURS ^*^ Mann-Whitney U test ^**^ Chi-squared test ^†^ Fisher’s exact test p-Values <0.05 were considered statistically significant. ESWL, extracorporeal shockwave lithotripsy; fURS, flexible ureteroscopy; PCNL, percutaneous nephrolithotomy; S-UAS, suction ureteral access sheath; T-UAS, traditional ureteral access sheath

Characteristic	S-UAS group (n = 50)	T-UAS group (n = 50)	p-Value
Stone analysis			0.58^**^
Calcium oxalate	21 (42%)	26 (52%)	
Calcium phosphate	18 (36%)	12 (24%)	
Uric acid	5 (10%)	5 (10%)	
Other	1 (2%), cystine	3 (6%), 2 struvite, 1 ammonium urate	
No stone analysis	5 (10%)	4 (8%)	
Complications	6 (12%)	8 (16%)	0.77^†^
Infection	4 (8%)	3 (6%)	
Retention	2 (4%)	2 (4%)	
Hematuria requiring irrigation	0	2 (4%)	
Renal colic from a retained ureter fragment	0	1 (2%)	
Follow-up imaging <6 months	24 (48%)	22 (44%)	0.84^†^
Imaging type	15 CT, 6 X-ray, 3 ultrasound	15 CT, 5 X-ray, 2 ultrasound	-
Presence of stone >4 mm on the side of the fURS	10/24 (42%)	9/22 (41%)	1.0^†^
Follow-up imaging <3 months	14 (28%)	13 (26%)	1.0^†^
Imaging type	12 CT, 1 X-ray, 1 ultrasound	12 CT, 1 X-ray	-
Presence of stone >4 mm on the side of the fURS	7/14 (50%)	6/13 (46%)	1.0^†^
Reintervention for persistent stone	9 (18%)	6 (12%)	0.58^†^
Reintervention type	7 fURS, 1 ESWL, 1 PCNL	5 fURS, 1 PCNL	-
Reintervention planned at the end of fURS	7/9 (78%)	4/6 (67%)	1.0^†^

Complications occurred in six patients (12%) in the S-UAS group and eight patients (16%) in the T-UAS group, which was not statistically significant (p = 0.77). S-UAS complications included four cases (8%) of UTIs and two cases (4%) of acute urinary retention. T-UAS complications included three UTIs (6%), two cases (4%) of acute urinary retention, two cases (4%) of hematuria requiring admission for irrigation, and one case (2%) of readmission with renal colic due to a retained ureteral fragment. No significant ureteral trauma from the UAS occurred in either group.

Follow-up imaging within six months of fURS was performed in 24 patients (48%) in the S-UAS group and 22 patients (44%) in the T-UAS group, with no significant difference between groups (p = 0.84). The most common follow-up imaging modality was a CT scan. Of those who underwent imaging, 10 out of 24 patients (42%) in the S-UAS group had a stone fragment larger than 4 mm, compared with nine out of 22 patients (41%) in the T-UAS group. There was no significant difference in the rate of persistent stone fragments within six months (p = 1.0) or within three months (p = 1.0), where seven out of 14 patients (50%) in the S-UAS group and six out of 13 patients (46%) in the T-UAS group had fragments larger than 4 mm.

Reintervention rates for persistent stones were similar between the groups (p = 0.58): nine patients (18%) in the S-UAS group and six patients (12%) in the T-UAS group. Reinterventions in the S-UAS group included seven fURS, one ESWL, and one PCNL, whereas in the T-UAS group, they included five fURS and one PCNL. Of the S-UAS patients requiring further procedures, seven out of nine (78%) were planned immediately after the initial fURS, which was similar to the T-UAS group, where four out of six patients (67%) were planned at the time of the initial procedure (p = 1.0).

Cost analysis

For this analysis, it was assumed that other factors, such as staffing and theater costs, were identical. Table 4 presents the costs to this single center for each piece of equipment included in the analysis. While the upfront cost of an S-UAS is higher than that of a T-UAS, baskets are more expensive than the S-UAS.

**Table 4:** Costs to the department for each piece of equipment used during fURS fURS, flexible ureteroscopy; S-UAS, suction ureteral access sheath; T-UAS, traditional ureteral access sheath

Equipment	Cost (£)
T-UAS	120
S-UAS	160
Standard basket	250

During the 50 S-UAS fURS cases, 14 required baskets and 36 did not. The 14 S-UAS cases with baskets cost £5,740 (14 × (160 + 250)), and the 36 cases without baskets cost £5,760 (36 × 160), resulting in a total equipment cost of £11,500 for the 50 S-UAS fURS, or an average of £230 per case. During the 50 T-UAS fURS cases, 45 required baskets and five did not. The 45 T-UAS cases with baskets cost £16,650 (45 × (120 + 250)), and the five cases without baskets cost £600 (5 × 120), resulting in a total equipment cost of £17,250 for the 50 T-UAS fURS, or an average of £345 per case. This cost analysis suggests that using S-UAS instead of T-UAS resulted in estimated cost savings of £115 per case, totaling £5,750 over 50 fURS procedures.

## Discussion

The primary endpoint of this study was to assess whether the rate of persistent stone fragments following fURS for renal stones was lower with S-UAS compared to T-UAS at three and six months postoperatively. Our results showed no difference in the rates of persistent stone fragments larger than 4 mm between the S-UAS and T-UAS groups. Secondary aims included evaluating whether S-UAS led to reduced operative times, fewer complications, lower reintervention rates, and cost savings due to reduced basket usage. Operative time trended longer in the S-UAS group, although this did not reach statistical significance (p = 0.099). No significant differences were observed in complications or reintervention rates, but basket usage was significantly lower in the S-UAS group (p < 0.001), and the cost analysis suggests this likely led to cost savings.

Previous studies assessing S-UAS have reported mixed results regarding stone-free rates at days 1 and 30 post-fURS [12,13,15]. Gauhar et al. [11] found a high stone-free rate with S-UAS, with 97.2% of patients having either no fragments or a single fragment <2 mm at 30 days post-fURS, though this study lacked a T-UAS comparison group. Most studies have not demonstrated significantly shorter operative times for S-UAS cases. Meta-analyses by Kadian et al. [15] and Xie et al. [21], as well as the multi-center randomized controlled trial by Zhu et al. [13], found no statistical difference in operative time between S-UAS and T-UAS. Liu et al. [12] suggested that operative time may actually trend longer for S-UAS cases. Studies generally indicate that S-UAS is associated with lower incidences of post-fURS fever but similar rates of other complications [12,13,15,21].

Reintervention rates following fURS with S-UAS are important to assess whether reduced persistent stone fragments translate into fewer additional procedures. Current evidence is mixed: Liu et al. [12] and Xie et al. [21] did not report reintervention rates; Zhu et al. [13] found no significant difference, while Kadian et al. [15] reported a lower reintervention rate with S-UAS. Regarding basket usage, Gauhar et al. [11] reported a 13% basket usage rate with S-UAS, and Zhu et al. [13] found lower basket usage with S-UAS compared to T-UAS. However, there is a lack of studies evaluating whether reduced basket use with S-UAS translates into cost savings, given the slightly higher upfront cost of S-UAS.

This study adds to the literature by including a cost analysis and assessing planned or completed reintervention up to six months after fURS. It also provides real-world data, with no upper stone size limit, inclusion of partial staghorn stones, and no exclusion for abnormal renal or ureter anatomy. While the stone-free rate was used as the primary endpoint, it could be argued that the reintervention rate would be a more clinically relevant measure. The power calculation was based on stone-free rates, suggesting a minimum of 39 patients per group; a reintervention-based endpoint would have required a larger sample size.

Several factors may explain the slightly longer operative time in the S-UAS group. First, S-UAS is a newer technology, and the learning curve for setting up suction and determining optimal fragment removal likely contributed to longer procedures. Future studies could mitigate this by including only surgeons with prior S-UAS experience. Second, the S-UAS group appeared to include more complex cases, with multiple stones, bilateral procedures, partial staghorn stones, and stones >2 cm, although these differences were not statistically significant. Liu et al. [12] suggested that S-UAS may slightly prolong fURS due to differences in stone elimination strategies: T-UAS typically lasers stones to <2 mm fragments to pass spontaneously, whereas S-UAS allows suction of slightly larger fragments, requiring additional suction time.

Limitations of this study include limited follow-up imaging, with fewer than 50% of patients in each group undergoing imaging within six months. This likely reflects clinical practice, as patients with low preoperative stone burden or uncomplicated procedures often do not receive early imaging. Those who did undergo follow-up imaging were often planned for reintervention or had complications, likely contributing to higher observed rates of persistent fragments. Imaging modalities varied, including CT scans, X-rays, and ultrasounds. Inclusion of larger stones (>2 cm) and partial staghorn stones may also have contributed to higher residual fragment rates.

Another limitation is the study design, with prospective S-UAS data and retrospective T-UAS data, which may introduce bias. Stone characteristics were not identical between groups, with the S-UAS group having more complex cases, likely reflecting the surgeon's selection of S-UAS for more challenging procedures. While there were no differences in infection rates between groups, two T-UAS patients were readmitted with hematuria requiring three-way catheter irrigation, whereas no S-UAS patients experienced this complication.

Reduced basket use with S-UAS may have benefits beyond cost savings. Basket usage carries risks, including breakage, ureteral abrasions or strictures, and, rarely, ureteral avulsion [22,23]. Although no basket-related complications occurred in this study, fewer baskets used during S-UAS may reduce these risks in larger cohorts.

Future research should focus on whether S-UAS reduces reintervention rates compared to T-UAS, as current literature is conflicting. Given the expected narrow difference in reintervention rates, adequately powered studies will need large sample sizes.

## Conclusions

This study found that S-UAS use during fURS for renal stones resulted in significantly less basket usage, leading to cost savings. Rates of persistent fragments, reintervention, and complications were similar between the S-UAS and T-UAS groups. Operative time trended longer in the S-UAS group, although this difference did not reach statistical significance. A large randomized controlled trial with more consistent follow-up imaging, specifically assessing whether S-UAS reduces reintervention rates compared to T-UAS, would be valuable to further evaluate the effectiveness of S-UAS.
